# Dipeptidyl peptidase‐4 inhibitors and aerobic exercise synergistically protect against liver injury in ovariectomized rats

**DOI:** 10.14814/phy2.14191

**Published:** 2019-09-08

**Authors:** Nagat Younan, Samah Elattar, Mira Farouk, Laila Rashed, Suzanne Estaphan

**Affiliations:** ^1^ Physiology Department, Faculty of Medicine Cairo University Cairo Egypt; ^2^ Histology Department, Faculty of Medicine Cairo University Cairo Egypt; ^3^ Biochemistry Department, Faculty of Medicine Cairo University Cairo Egypt; ^4^ ANU Medical School Australian National University Canberra Australian Capital Territory Australia

**Keywords:** DPPi, exercise, fatty liver, ovariectomy

## Abstract

Menopause increases the risk of non‐alcoholic fatty liver disease (NAFLD). We investigated the effect of incretin and/ or exercise on the hepatic fat accumulation in ovariectomized rats. Rats were divided into five groups: Group 1: Control rats, Group 2: Ovariectomized rats, Group 3: Ovariectomized rats + Dipeptidyl peptidase‐4 inhibitor (DPPi) (30 mg/kg/day, orally), Group 4: Ovariectomized rats + swimming, and Group 5: Ovariectomized rats + swimming + DPPi. After 6 weeks, Alanine aminotransferase (ALT), glucose, insulin, HOMA IR (Homeostatic Model Assessment for Insulin Resistance), FFA (free fatty acids), Tumor necrosis factor alpha (TNF *α*), IL6, IL1B levels were measured in blood. The livers were collected for Hematoxylin and eosin (H&E) examination and evaluation of hepatic gene expression of SREBP (sterol regulatory element‐binding protein1c), PPAR *α* (peroxisome proliferator‐activated receptor alpha), ACC 1 (acetyl‐CoA carboxylase), LC3 (microtubule‐associated protein 1 light chain 3), SIRT (sirtuin), hepatic triglycerides, IL6, IL10, caspase 3 and AMPK (adenosine monophosphate‐activated protein kinase). A significant increase in ALT level and area of liver tissue defects with a significant increase in glucose HOMA IR, serum FFA, IL6, IL1B, TNF *α*, liver TGs (triglycerides), inflammation, apoptosis, SREBP1c, ACC1 were found in ovariectomized rats as compared to control group with a significant decrease in PPAR *α*, LC3, AMPK and SIRT1. DPPi treated rats with and without exercise showed a significant improvement in ALT and area of liver tissue defects, inflammation and apoptosis and serum IL6, IL1B, TNF *α*, FFA, liver LC3, SIRT1, AMPK, TGs, PPAR *α*, ACC1 and SREBP1c as compared to the ovariectomized group. Findings from the study confirm the derangement of fat metabolism in the ovariectomized rats and showed that incretin‐based therapy and exercise synergistically improved liver fat metabolism, achieved significant beneficial metabolic effects and offer full protection against NAFLD.

## Introduction

There are many changes after menopause that are assumed to affect the function of the liver and mediate the development of liver disease (Brady 2015). Ovariectomy in female rats is a commonly used animal model for the assessment of menopause problems (Yoo et al. 2016).

Decreased estrogen during menopause increases insulin resistance, causes dyslipidemia and disturbs the balance between the production and use of fatty acids resulting in steatosis with fat accumulation in the liver with development of non‐alcoholic fatty liver (NAFLD) (Mauvais‐Jarvis et al. 2013). The main mechanism for the development of steatosis and NAFLD is due to both increased lipid storage and decreased lipid removal (Reddy and Rao 2006; Musso et al. 2009). Ovariectomy inhibits peroxisome proliferator‐activated receptor alpha (PPAR*α*), E2 has been demonstrated to induce the formation of a prostaglandin D2 metabolite capable of acting as a ligand for PPAR (Ma et al. 1998). which is involved in peroxisomal fatty acid β‐oxidation and enhances acetyl‐CoA carboxylase (ACC) that catalyzes hepatic fatty acid synthesis (Fischer et al. 2003). This disruption in enzymatic level enhances triglycerides synthesis and suppresses their oxidation in the liver of hormone deprived rats.

Sterol regulatory element‐binding protein1c (SREBP1c) transcription factor is an essential regulator for hepatic fat metabolism and affects hepatic genes coding for fatty acid biosynthesis (Shimomura et al. 1999; Pettinelli et al. 2009). In ovariectomized rats reduced SREBP1c leads to lipid accumulation in their livers (Paquette et al. 2008)

Autophagy is a degradation process for cells to turn over organelles and old molecules. Disruption of the autophagy results in accumulation of unwanted molecules including fat droplets. Autophagy has also been implicated in cell death via apoptosis. Autophagosome formation and maturation involves a number of proteins, including microtubule‐associated protein 1 light chain 3 (LC3)‐I, which is an ubiquitin‐like protein that conjugates with phosphatidylethanolamine to form LC3‐II (Kluge et al. 2013). It is suggested that hormone deficiency during menopause may affect this process adding to the failure of getting rid of fat droplets leading to their accumulation (Yao et al. 2018).

Estrogen may play a role in post‐menopausal liver function by promoting fatty acid oxidation and increasing the efficiency of triglyceride export out of the liver (Zhu et al. 2013)

Incretins are intestinal hormones that enhance glucose‐stimulated insulin secretion (Baggio and Drucker 2007). As circulating Glucagon‐like peptide 1 (GLP‐1) is rapidly degraded by the enzyme dipeptidyl peptidase‐4 (DPP‐4), DPP‐4 inhibitors (DPPi) have been developed (Ahren and Schmitz 2004). Some studies suggest that incretin may be of new therapeutic value for NAFLD (Lee et al. 2012). Although there is no clear evidence that incretin secretion is inhibited in ovariectomized rats, many studies tried the beneficial effect of incretin based therapy to improve the hormone deficiency symptoms in the postmenopausal period (Ma et al. 2013; Lu et al. 2015).

Recent systematic reviews of randomized controlled trials indicate that exercise interventions improve glycemic control in subjects with impaired glucose tolerance and type 2 diabetes mellitus (T2DM) (Yoon et al. 2013; Rohling et al. 2016), and reduce hepatic fatty infiltration in patients with NAFLD (Golabi et al. 2016; Orci et al. 2016).

Aim: To investigate the effect of DPPi (sitagliptin) treatment with or without exercise on hepatic fat accumulation and prevention of development of fatty liver in ovariectomized rats.

## Material and Methods

### Experimental Animals

A total of 30 adult female albino rats (150–200 g) were used. The protocol and all the procedures were approved by the Institutional Animal Care and Use Committee of Cairo University (IACUC). The rats were purchased from the Laboratory Animal House Unit where they received veterinary care. The rats were housed in wire mesh cages at comfortable temperature about 23 ± 1°C, under a normal light‐dark cycle and had free access to rat chow and water.

#### Experimental protocol

Rats were randomly divided into five groups (each *n* = 6 rats).

Group 1: Control sham operated rats, Group 2 (OVX): Ovariectomized rats, Group 3 (OVX + DPPi): Ovariectomized rats treated with dipeptidyl peptidase‐4 inhibitor (DPPi) ** (30mg/kg/day, orally) for 6 weeks starting from the day after the operation, Group 4 (OVX + Ex): Ovariectomized rats + swimming*, and Group 5 (OVX + DPPi+Ex): Ovariectomized rats + swimming + DPPi.

*Exercise program was in the form of swimming for 60 min per day, 5 days per week for 6 weeks, without attaching weights to the animals starting from the day after the operation (Falcai et al. 2015).

** Sitagliptin 50 mg, a tablet was dissolved in 10 mL of distilled water, each rat was given 1 mL of the solution by oral gavage.

Animals were anaesthetized (ketamine 80 mg/kg and xylazine 10 mg/kg, ip) and underwent either ovariectomy (Khajuria et al. 2012) (group 2, 3, 4, 5) or sham operation (group 1).

#### Measurements

At the end of 6‐week‐period of the experiment, and after an overnight fasting, blood samples were collected through retro‐orbital route using heparinized capillary tubes to evaluate alanine transaminase (ALT), Glucose, insulin, Homeostatic Model Assessment of Insulin Resistance (HOMA IR), free fatty acids (FFA), tumor necrosis factor alpha (TNF *α*), interleukin 6 (IL6), and interleukin 1B (IL1B) levels.

Rats were culled by cervical dislocation and the liver was excised and processed within 2 min of removal from the body. Liver tissue samples were collected from the right lobe for H&E examination and evaluation of hepatic gene expression of sterol regulatory element‐binding protein (SREBP), PPAR *α*, ACC 1, LC3, NAD‐dependent deacetylase sirtuin‐1 (SIRT) and 5′ adenosine monophosphate‐activated protein kinase (AMPK), hepatic triglycerides, IL6, IL10 and caspase 3.

#### Concise methods

##### Biochemical methods

Conventional colorimetric method was used to measure serum glucose and triglyceride (TG) levels using QuantiChrom TM assay kit (Corporate Place; Hayward; USA).

Insulin levels were measured using an ELISA kit supplied by (DRG, USA) in accordance with the manufacturers’ instruction.

HOMA index was calculated using the equation provided by Matthews et al. (1985).

The ALT was measured using a kit from Randox Laboratories (England) using colorimetric methods.

Serum free fatty acid was detected using a kit supplied by Mybiosource, USA according to supplied protocol.

Measurement of IL6, IL10, IL1b, TNF *α* and caspase 3:

They were measured in serum and in tissue homogenate using a kit supplied by Ray Biotech, USA according to the manufacturer's instruction.

#### Quantitative real‐time PCR

Real‐time PCR was performed for quantitative gene expression of (PPAR *α*, SREBP1, LC3, ACC1& sirt1) using RNeasy purification reagent (Qiagen, Valencia, CA), reverse transcription reaction (Superscript II, Gibco Life Technologies, Grand Island, NY, USA) and Applied Biosystems with software version 3.1 (StepOneTM, USA). The primers used are shown in Table 1 (Dawood et al. 2018)

**Table 1:** Primer sequences of studied genes.

Gene	Sequence
SIRT1	Forward primer:5′‐ GATCTCCCAGATCCTCAAGCC‐3′
	Reverse primer:5′‐ CACCGAAGGAACTACCTGAT‐3′
SREBP1	Forward: 5‐CAT CAA CAACCA AGA CAG TG‐3
	Reverse: 5‐GAA GCA GGA GAA GAGAAG C‐3
ACC1	Forward primer:5′‐ CGGCAACAAACAAGGG‐3
	Reverse primer:5′‐ CGTTACAACCAGGAAGCC‐3
LC3	Forward primer:5′‐ GGT CCA GTT GTG CCT TTA TTG A‐3′
	Reverse primer:5′‐ GTG TGT GGG TTG TGT ACG TCG‐3′
PPAR	Forward primer: 5′‐ ACTCCACCTGCAGAGCAACCA ‐ 3′
	Reverse primer:5′‐ TAGATCTCCTGCAGTAGCGGG −3′
Beta‐actin	Forward primer: 5′ ‐ GACGGCCAGGTCATCACTAT −3′
	Reverse primer: 5' ‐ CTTCTGCATCCTGTCAGCAA −3'

Detection of AMPK protein using Western Blot technique (using V3 Western Workflow™ Complete System, Bio‐Rad® Hercules, CA) and antibodies for AMPK and beta‐actin were supplied by Thermoscientific (Rockford, Illinois, USA) (Elattar et al. 2017)

##### Histological examination

For histological study, samples from the the right lobes of the liver were dissected and immediately fixed in 10% formol saline for 48 h. Sections were then processed in ascending grades of alcohol (70%, 90%, 100%), cleared in xylol, and embedded into paraffin blocks.

Serial sections were then cut at 5 *µ*m thickness and routinely stained with H&E. Sections were photographed at Histology department, faculty of Medicine. Sections were also examined using Leica Qwin 500 Image analysis. To evaluate areas of tissue defect in each sample, total measuring frame (as seen on computer screen) was measured. Then, each tissue defect in each serial field was separately measured and the areas were added together to get one result for one field. Ten nonoverlapping serial fields were measured in each sample to cover most of the tissue.

Histomorphometry was done and included the five studied groups. The parameters measured were:

1. Measurement of areas of tissue defect, as total area of all defects in serial nonoverlapping fields.
2. Measure of diameter of hepatocytes.
3. Count of cells seen as clearly apoptotic, in serial nonoverlapping fields for each sample.
4. Count of cells with evidence of margination of chromatin and loss of nucleoli.

#### Statistical methods

The collected data were statistically analyzed using SPSS. We calculated the mean values and their standard deviation. The difference among the mean values and multiple comparison between pairs of groups was estimated using Analysis of variance (ANOVA) Post Hoc test. Pearson Correlation was done to assess the relation between variables. Significance was assumed at *P* < 0.05.

## Results

### Histopathological examination

**Figure 1:**
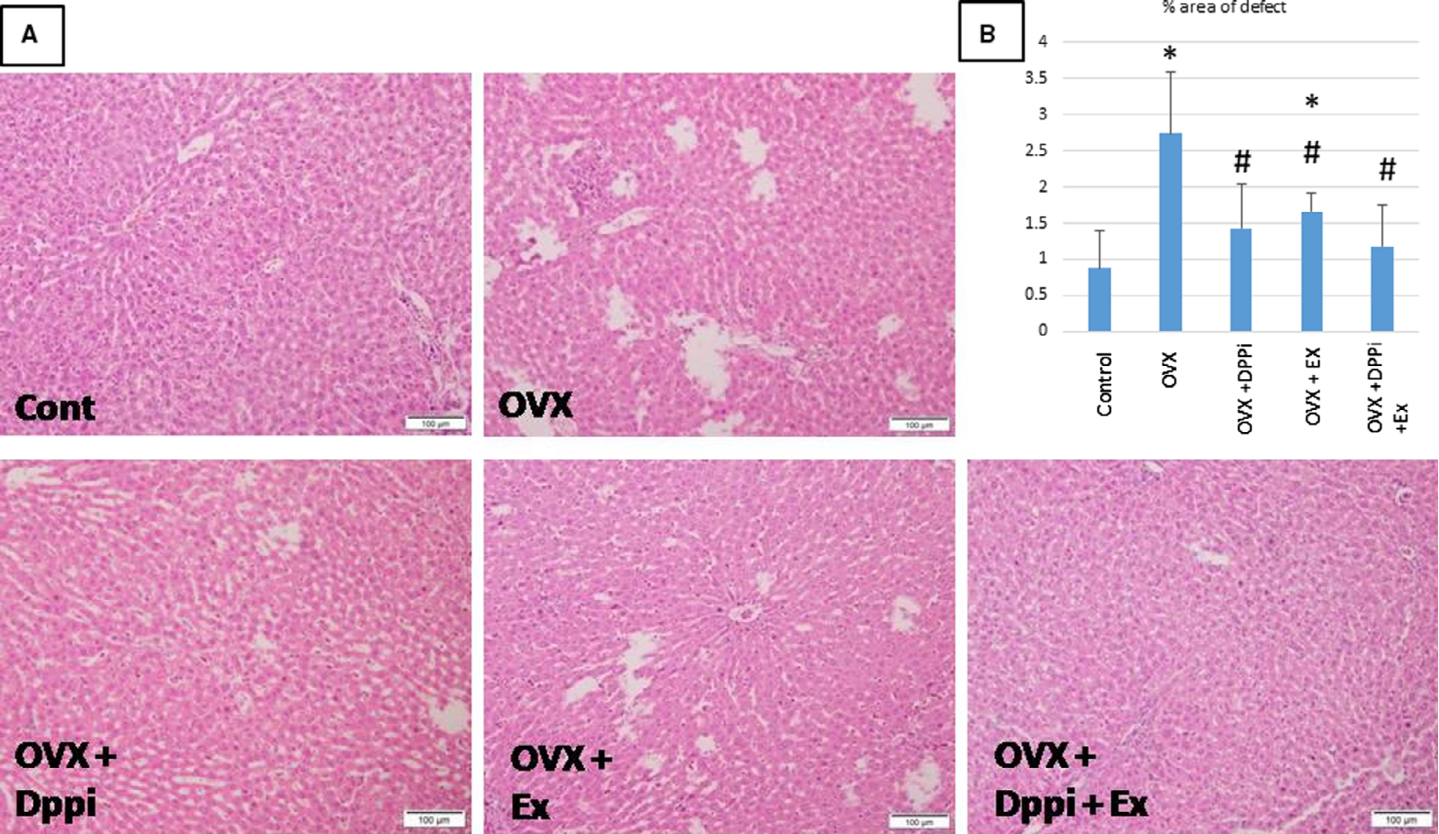
(A) Overview of area defects of liver tissue in the different studied groups. Control: shows continuous sheets of cells over wide areas, with no tissue defects (at low magnification). Note the portal tract at lower left part of the field. OVX: shows variable sized disseminated patches of tissue loss. One area (upper left) shows apoptotic cells with inflammatory infiltration. OVX + DPPi: shows minimal scattered areas of tissue loss. OVX + Ex: shows disseminated patches, but considerably smaller than the OVX group. OVX + DPPi + Ex: is very similar in appearance to control sections, with many wide fields of continuity of sheets of hepatocytes, properly arranged into the classic hepatic lobules. (B) Chart showing the percentage of the area of liver tissue defect in all groups as percentage to the total area of the field. Values are represented as mean ± SD. *Statistically significant compared to corresponding value in control group (*P* < 0.05). #Statistically significant compared to corresponding value in ovariectomized (OVX) group (*P* < 0.05).

**Figure 2:**
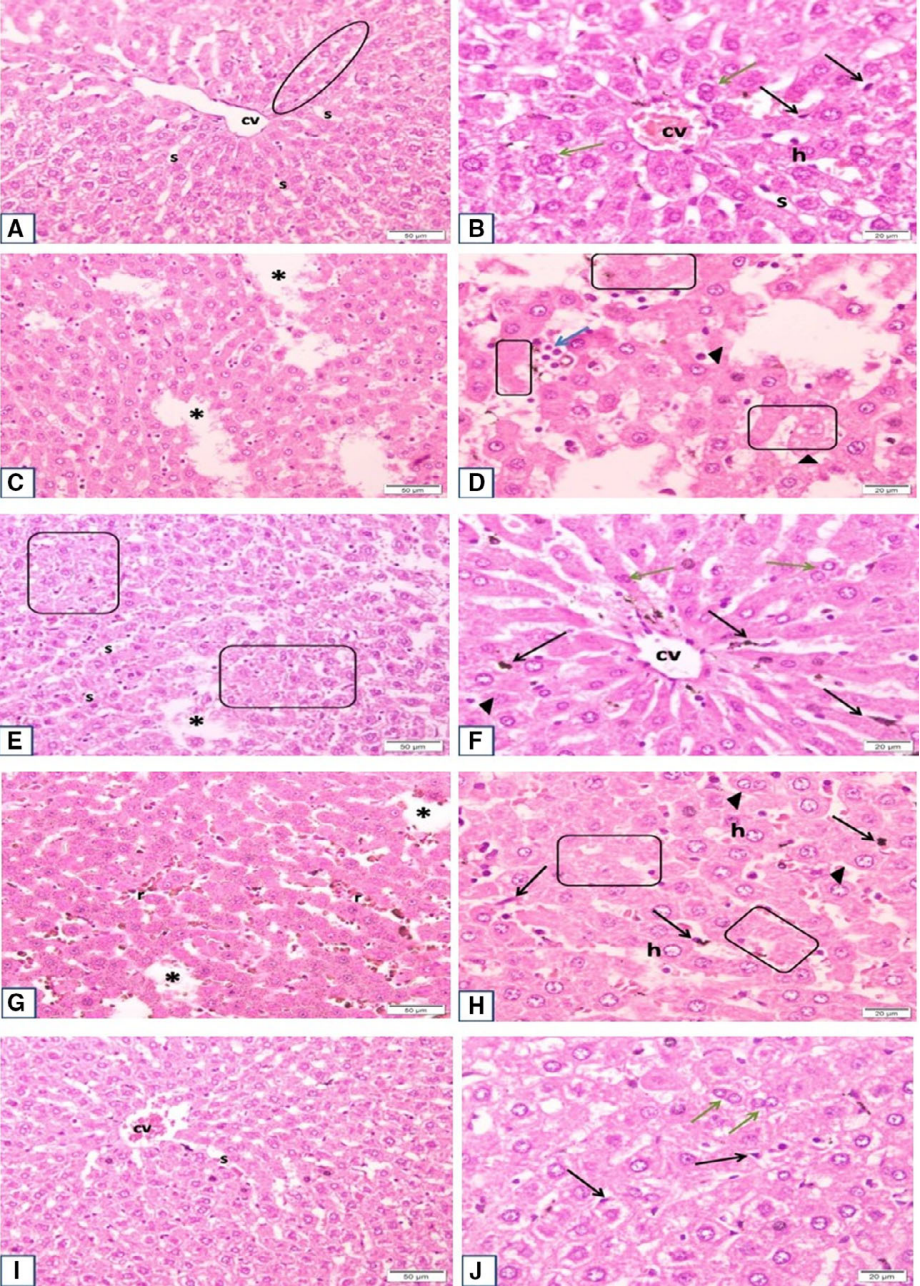
Plate shows sections of the different experimental groups at low and high magnification: (A and B) Control Group sections show normal architecture of hepatic lobule with cords of hepatocytes radiating from the central vein, with sinusoids in between. Sheets of hepatocytes and intervening sinusoids extend over wide areas, with no tissue defects. Hepatocytes show large vesicular nuclei. Some are binucleated. Cytoplasm stains acidophilic. Von Kuppfer cells are seen within and lining the sinusoids. (C and D) OVX sections show many variable sized areas of tissue defect that extend all over the sections. Apoptotic cells and inflammatory infiltrate are seen. Many nuclei of hepatocytes are shrunken and darkly stained. Other parts are devoid of nuclei with pale acidophilic staining and vacuolation. Mononuclear infiltrate is more evident in areas of cell debris. Occasional giant cells can be seen. However, there is no evidence of overall increase of von Kuppfer cells. (E and F) OVX + DPPi group: shows evident decrease in areas of tissue defect, with restoration of normal spreading of hepatocytes along the sinusoids, however, scattered regions have small sized hepatocytes with pale nuclei, markedly vacuolated cytoplasm and irregular outline of cells. Other areas are more regular with few apoptotic cells and normal population of von Kuppfer cells. Binucleated cells are evident. (G and H) OVX + Ex group shows less marked decrease in overall areas of tissue defects (when compared to drug treated group). Higher magnifications show prominent increase in von Kuppfer cell population, lining and within the sinusoids, with hyperemia. Hepatocyte cell vacuolation is also decreased. Most nuclei are vesicular and cells are of normal size. Only small regions show lack of nuclei or dark nuclei with deep stained cytoplasm denoting degeneration. (I and J) OVX + DPPi + Ex group shows minimal area defects, with overall picture similar to control group. Most cells are of average normal size, with pale nuclei, pale acidophilic staining and minimal or few vacuolations, von Kuppfer cell population is normal, and sinusoids show no hyperemia. Thus, hepatocytes of this group cover most areas, and are of normal pattern and size. cv, central vein; s, sinusoids; oval area, rows of hepatocytes; h, hepatocytes (normal size & vesicular nuclei); black arrow, von Kupffer cells; asterisk*, area defects; arrowheads, apoptotic cells; blue arrow, inflammatory infiltrate; green arrow, binucleated hepatocytes; boxed area, small irregular & vacuolated cells; r, RBCs (hyperemia).

**Figure 3:**
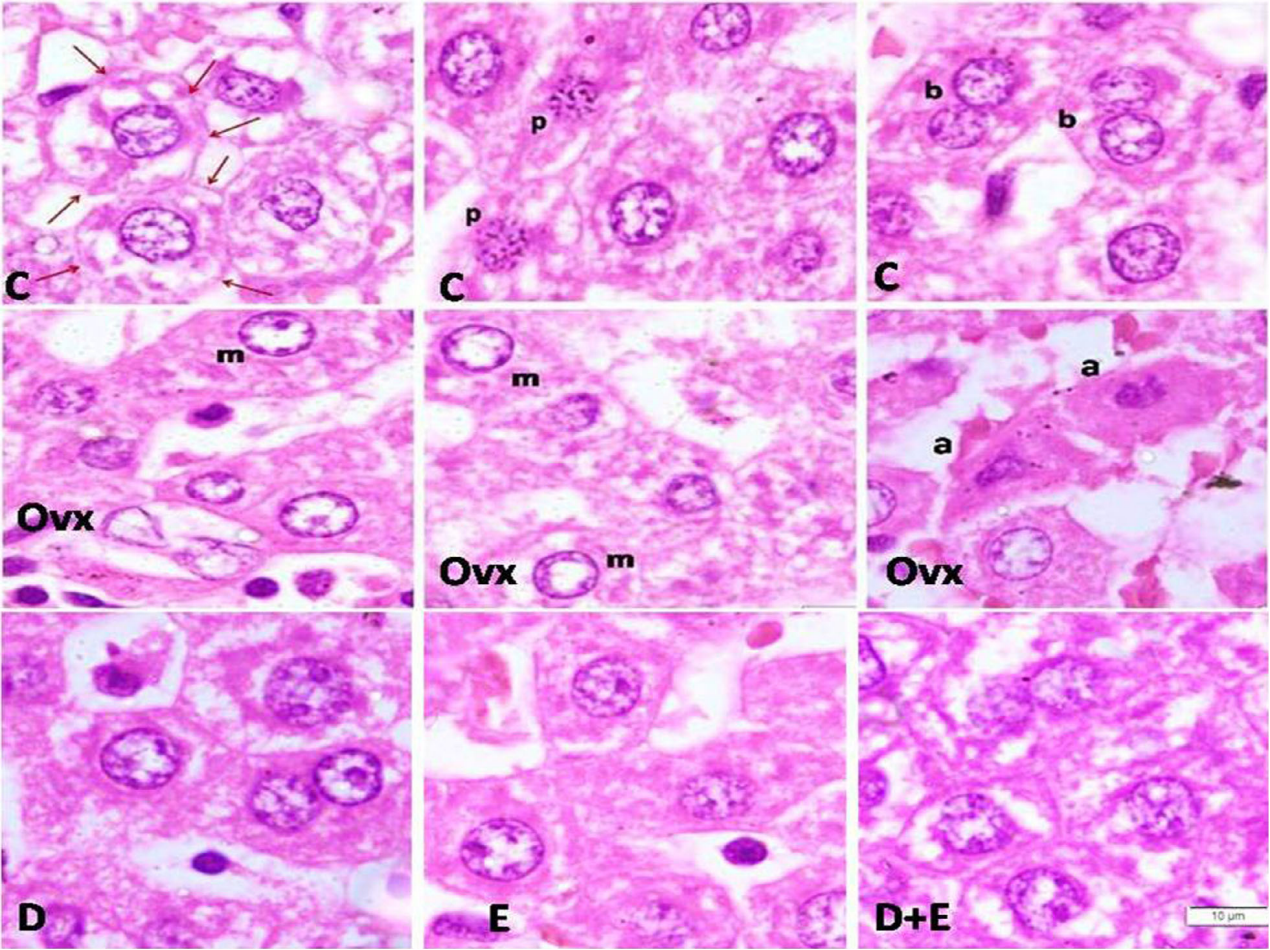
Upper row: Magnified and cropped sections from control (c) group show vacuolation within the cytoplasm of intact cells with complete cell membranes (denoted by red arrows), most probably denoting glycogen storage. Nuclei of the cells show the normal pale vesicular appearance with two or three nucleoli, characterstic of the hepatocytes. Sections also show evidence for many binucleated cells (b), and cells in prophase (p). Middle row shows sections from Operated (op) group: many cells have nuclei with marginated chromatin (m) and loss of pattern; others nuclei are lost. Inflammatory cells are seen, as well as distorted outlines and loss of architecture of cells. Also, there are deep eosinphilic distorted cells, with abnormal small dark nuclei, denoting changes of apoptosis (a). Last row: shows sections from the three treated groups. DPPI group (D) shows all normal vesicular nuclei and binucleated cells. Ex group (E) shows improvement, with only few cells not showing nuclei. DPPI + Ex group (D + E) shows picture similar to control, with restoration of vacuolation in intact cells, indicative of glycogen storage.

As shown from Figures 1, 2, 3, the sections of liver tissues obtained from the control group showed normal architecture of hepatic lobule. Sheets of hepatocytes and intervening sinusoids extend over wide areas, with no tissue defects. Hepatocytes show large vesicular nuclei. Some are binucleated. Cytoplasm is acidophilic. Von Kuppfer cells are seen within and lining the sinusoids (Figs. 1, 2‐A and B and 3).

Sections from the liver taken from the ovariectomized rats (group 2) showed many variable sized areas of tissue defect, together with apoptotic cells and inflammatory infiltrate. These areas extend all over the sections. Many nuclei of hepatocytes are shrunken and darkly stained. Other parts are devoid of nuclei with pale acidophilic staining and vacuolation. Mononuclear infiltrate is more evident in areas of cell debris. Occasional giant cells can be seen. However, there is no evidence of overall increase of von Kuppfer cells (Figs. 1, 2‐C and D and 3).

Sections obtained from the liver of the group of rats that received DPPi for 6 weeks after ovariectomy (group 3) showed an evident decrease in areas of tissue defect, with restoration of normal spreading of hepatocytes. However, scattered regions had small sized hepatocytes with pale nuclei, markedly vacuolated cytoplasm and irregular outline of cells. Other areas were more regular with few apoptotic cells and normal population of von Kuppfer cells. Binucleated cells were evident (Figs. 1, 2‐E and F and 3).

Sections from the livers of exercise trained rats after ovariectomy (group 4) showed less marked decrease in overall areas of tissue defects (when compared to drug treated group). Higher magnifications show prominent increase in von Kuppfer cell population lining and within the sinusoids, with hyperemia. Hepatocyte cell vacuolation was also decreased. Most nuclei were vesicular and cells were of normal size. Only small regions showed lack of nuclei or dark nuclei with deep stained cytoplasm denoting degeneration (Figs. 1, 2‐G and H and 3).

Sections from rats that received DPPi and underwent exercise training program for 6 weeks after ovariectomy (group 5) showed minimal area defects, with overall picture similar to control group. Most cells were of average (normal) size, with pale nuclei, pale acidophilic staining and minimal or few vacuolations. The von Kuppfer cell population was normal and sinusoids showed no hyperemia. Thus, hepatocytes of this group covered most areas, and were of normal pattern and size (Figs. 1, 2‐I and J and 3).

**Figure 4:**
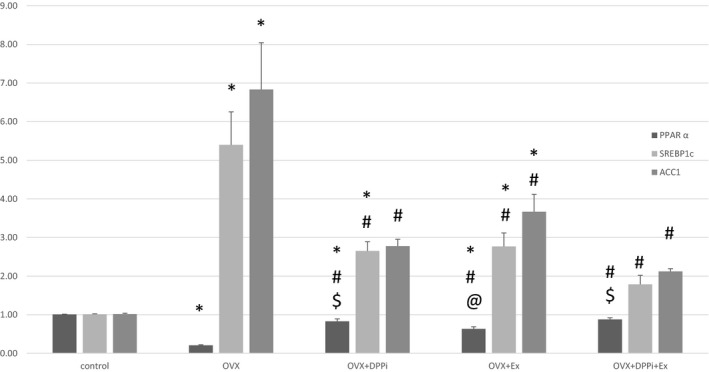
Chart showing hepatic gene expression of PPAR *α*, ACC1 and SREBP1c in different studied groups. Values are represented as mean ± SD. *Statistically significant compared to corresponding value in control group (*P* < 0.05). #Statistically significant compared to corresponding value in ovariectomized (OVX) group (*P* < 0.05). @Statistically significant compared to corresponding value in DPPi treated (OVX + DPPi) group (*P* < 0.05). $Statistically significant compared to corresponding value in exercise (OVX + Ex) group (*P* < 0.05).

Table 2 and Figures 1 and 4 demonstrate that ovariectomized rats showed a significant increase in ALT level, liver areas of defects, glucose and HOMA IR, serum FFA, liver TGs, SREBP1c, ACC1 and a significant decrease in PPAR *α* while showing no significant change in insulin levels as compared to the control group. DPPi treated rats with and without exercise showed a significant improvement in ALT level and area of liver tissue defects, HOMA IR, serum FFA, liver TGs, PPAR *α*, ACC1 and SREBP1c as compared to the ovariectomized group and showed no significant difference in these parameters except PPAR *α* and SREBP1c as compared to the control group while these groups exerted a significant higher PPAR *α* level as compared to the exercise group, while only the combined DPPi treatment with exercise group showed no significant difference in PPAR *α* and SREBP1c as compared to control group. Trained rats with no drug treatment also showed a significant improvement in serum ALT, FFA, liver TGs and area of liver tissue defects as compared to the ovariectomized group with no significant difference in ALT, glucose, insulin and HOMA IR as compared to control group but had a significant increased liver tissue defects area as compared to control. The combined DPPi treatment with exercise group showed a significant lower glucose level as compared to the ovariectomized group and the exercise only group. The three groups of rats treated with DPPi and/or exercise showed no significant difference in ALT, insulin, HOMA IR, serum FFA, liver TGs, ACC1, SREBP1c and liver tissue defects area as compared to each other (Table 2 and Figs. 1 and 4).

**Table 2:** Serum levels of ALT, glucose, insulin and calculated HOMA IR, liver content of TGs and serum FFA in the studied groups.

	Control	OVX	OVX + DPPi	OVX + Ex	OVX + DPPi+Ex
ALT (U/L)	12 ± 2.83	53 ± 10.44*	21 ± 2.16†	25.67 ± 3.51†	18.67 ± 1.53†
glucose (mmol/L)	6.60 ± 0.42	8.93 ± 1.02*	7.45 ± 0.39	8.57 ± 0.42	6.67 ± 0.57†‡
insulin (mIU/L)	7.85 ± 0.35	8.80 ± 1.23	8.23 ± 0.57	8.14 ± 0.78	8.20 ± 0.11
HOMA IR	2.3 ± 0.04	3.47 ± 0.42*	2.72 ± 0.23†	3.09 ± 0.14	2.43 ± 0.2†
TGs (mg/g protein)	11.4 ± 1.7	36.53 ± 6.29*	19.48 ± 2.06†	22.13 ± 2.67†	12.93 ± 0.4†
FFA (ng/mL)	13.75 ± 0.21	34.00 ± 6.25*	20.63 ± 0.9†	22.77 ± 4.73†	13.87 ± 0.35†

Values are represented as mean ± SD. ALT, Alanine transaminase; HOMA IR, Homeostatic Model Assessment of Insulin Resistance; TGs, hepatic triglycerides; FFA, serum free fatty acids.

* Statistically significant compared to corresponding value in control group (*P* < 0.05).

^†^ Statistically significant compared to corresponding value in ovariectomized group (*P* < 0.05).

^‡^ Statistically significant compared to corresponding value in exercise (OVX + Ex) group (*P* < 0.05).

**Figure 5:**
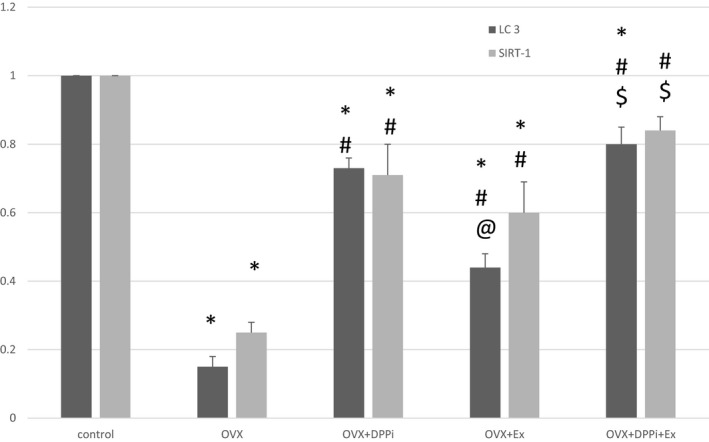
Chart showing hepatic gene expression of LC3 and SIRT1 in different studied groups. Values are represented as mean ± SD. *Statistically significant compared to corresponding value in control group (*P* < 0.05). #Statistically significant compared to corresponding value in ovariectomized (OVX) group (*P* < 0.05). @Statistically significant compared to corresponding value in DPPI treated (OVX + DPPi) group (*P* < 0.05). $Statistically significant compared to corresponding value in exercise (OVX + Ex) group (*P* < 0.05).

**Figure 6:**
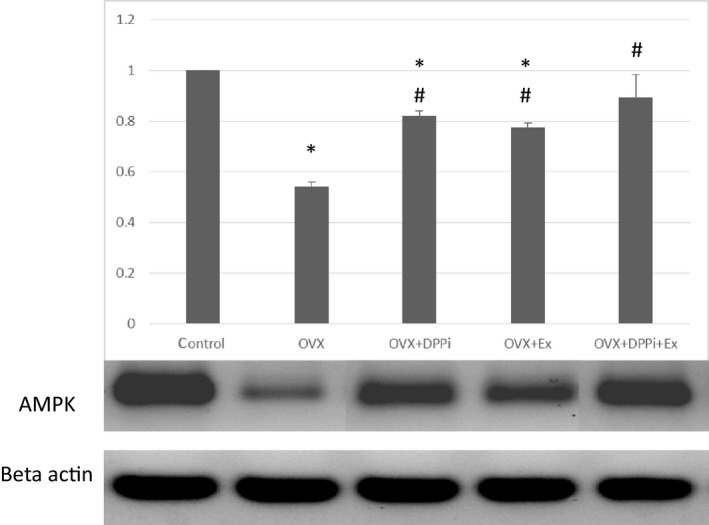
The results of western blot revealed that the expression of AMPK was low in ovariectomized rats (OVX) as compared to control. Expression of AMPK increased in OVX rats that received DPPi and/or underwent exercise training. Beta‐actin was used as a loading control. The chart shows values as mean ± SD. *Statistically significant compared to corresponding value in control group (*P* < 0.05). #Statistically significant compared to corresponding value in ovariectomized (OVX) group (*P* < 0.05).

Ovariectomized rats showed a significant increase in serum IL6, IL1B, TNF *α*, liver IL6, caspase 3 and a significant decrease in liver IL10, LC3, AMPK and SIRT1 as compared to control group. DPPi treated rats with and without exercise and trained rats without DPPi treatment showed a significant improvement in serum IL6, IL1B, TNF *α*, liver IL6, IL10, caspase3, LC3, SIRT1 and AMPK as compared to the ovariectomized group and showed no significant difference in liver IL6, IL10, caspase 3, AMPK as compared to each other. DPPi treatment with or without exercise induced a significant decrease in serum IL6, TNF *α* level and a significant increase in liver LC3 as compared to the exercise group. The combined DPPi treatment with exercise group showed a significant lower IL1B as compared to rats treated with DPPi only and rats which underwent exercise training only. DPPi treatment with or without exercise showed no significant difference in serum IL6, liver IL6, IL10 and caspase 3 levels as compared to the control group. Only the combined DPPi treatment with exercise group showed no significant difference in IL1B, liver IL10, SIRT1 and AMPK as compared to the control group and a significant higher liver SIRT1 as compared to rats which underwent exercise training only (Table 3 and Figs. 5 and 6).

**Table 3:** Shows serum level of IL6, IL1B and TNF*α* and liver tissue level of IL6, IL10 and caspase3.

	Control	OVX	OVX + DPPi	OVX + Ex	OVX + DPPi+Ex
Serum IL6 (pg/mL)	13.6 ± 1.27	85.63 ± 8.64*	29 ± 5.46†§	47.93 ± 3.73*†§	21.50 ± 1.87†§
Serum IL1B (pg/mL)	39.9 ± 3.82	137.9 ± 3.92*	69.43 ± 6.77b*†	75.23 ± 5.42*†	49.43 ± 4.25†‡§
Serum TNF*α* (pg/mL)	14.45 ± 1.34	78.7 ± 4.33*	27.83 ± 2.49*†§	43.9 ± 3.4*, †, ‡	25.67 ± 3.25*†§
Liver IL6 (pg/mg protein)	15.85 ± 2.33	91.77 ± 14.33*	39.13 ± 11.48†	42.07 ± 10.22†	23.2 ± 4.84†
Liver IL10 (pg/mg protein)	130.1 ± 2.4	67.43 ± 3.82*	101.4 ± 9.7*†	97.53 ± 10.86*†	107.47 ± 8.92†
Liver caspase3 (ng/mg protein)	2.15 ± 0.2	9.5 ± 1.91*	4.7 ± 0.36†	5.07 ± 1.15†	3.23 ± 0.32†

Values are represented as mean ± SD. IL, interleukin; TNF*α*, tumor necrosis factor alpha.

* Statistically significant compared to corresponding value in control group (*P* < 0.05).

^†^ Statistically significant compared to corresponding value in ovariectomized (OVX) group (*P* < 0.05).

^‡^ Statistically significant compared to corresponding value in DPPI treated (OVX + DPPi) group (*P* < 0.05).

^§^ Statistically significant compared to corresponding value in exercise (OVX + Ex) group (*P* < 0.05).

**Figure 7:**
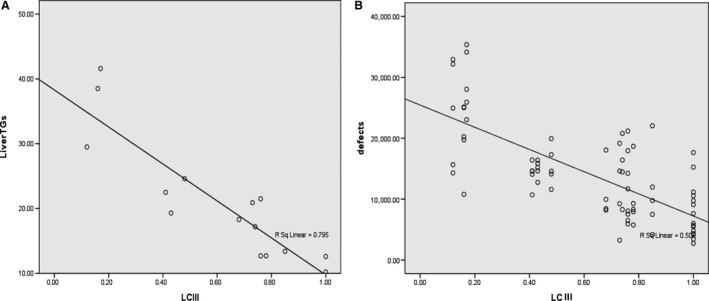
Overall correlation between LC III, liver TGs, and liver defect areas. Among all the studied groups, there was a significant negative correlation between LC3 and liver TGs (*r* = −0.902, *P* ≤ 0.001) [A], LC3 was significantly negatively correlated with liver defect areas (*r* = −0.712, *P* ≤ 0.001) [B].

Among all the studied groups, there was a significant negative correlation between LC3 and liver TGs, LC3 showed also a significant negative correlation with liver defect areas (Fig. 7).

## Discussion

In the present study, areas of tissue defect with apoptotic cells and inflammatory infiltrate were observed in the ovariectomized rat group with a significant elevation in liver enzyme and serum free fatty acids and hepatic triglycerides, inflammation and apoptosis. The glucose homeostasis was mildly disturbed, however, the reported values were considered within the normal non diabetic range.

Decreased estrogen during menopause disturbs the balance between the production and use of fatty acids resulting in fat accumulation in the liver with development of NAFLD (Mauvais‐Jarvis et al. 2013).

At the cellular level, ovariectomy disrupts enzymatic activity that is involved in lipid metabolism, PPAR*α* and Acetyl‐CoA carboxylase (ACC). This disruption in enzymatic level enhances triglyceride synthesis and suppresses their oxidation in the liver of hormone‐deprived rats (Shimomura et al. 1999).

DPPi treatment in ovariectomized rats for 6 weeks markedly preserved the hepatic architecture and protected the liver from injury and fat accumulation. Fat accumulation in liver was also prevented in ovariectomized rats by exercise training.

This was significantly proved through the histological examination and biochemical measurements of the liver tissue of the DPPi treated as well as exercise trained groups of ovariectomized rats that showed an evident decrease in areas of tissue defect, with restoration of normal spreading of hepatocytes as well as a significant decrease in the level of hepatic triglyceride content, inflammation and apoptosis and the serum liver enzyme ALT.

The liver highly expresses DPP‐4 (Mentzel et al. 1996), and it is suggested that incretin is highly involved in the regulation of hepatic metabolism. Tushuizen et al. (2006) reported that exenatide (GLP‐1 receptor agonist) therapy reduced hepatic fat content and improved liver functions.

The mechanism underlying this protection was studied and suggested to involve many pathways. First, DPPi increases hepatic insulin signaling and sensitivity (Gupta et al. 2010).

In the present study HOMA IR was significantly reduced after DPPi treatment as compared with the ovariectomized rats. DPPi treatment and exercise together enhance insulin sensitivity and bring values to be comparable with the control values. Park et al. (2010) reported that exendin‐4 therapy improves hepatic insulin signaling by increasing insulin receptor substrate‐2 tyrosine phosphorylation in diabetic rats fed with high fat diets. Also, muscle contraction increases AMPK activity, that deactivates RabGAP (Rab GTPase‐activating protein) TCB1D1, and enhances glucose transporter 4 (GLUT4) translocation to the cell membrane, this increases glucose uptake and improves insulin sensitivity (Bird and Hawley 2016). Improving insulin sensitivity has a great role in improving fat metabolism by the hepatocytes and preventing fat accumulation and damage (Utzschneider and Kahn 2006).

Second, incretin also has an antiinflammatory action and the inflammatory markers were found to be significantly reduced after DPPi treatment as compared with the ovariectomized rats.

Similarly, Shah and coworkers reported that DPP‐4 inhibitors attenuate macrophage proinflammatory activity (Shah et al. 2011). This action of DPPi may be by inhibiting DPP‐4 and potentiating incretin activity or directly through inhibition of protein kinase C (PKC) activity (Dai et al. 2014) and adenosine deaminase in macrophages (Shah et al. 2011). Exercise training also modulated the inflammatory response in the liver of ovariectomized rats and synergistically with DPPi treatment decreased the proinflammatory markers.

Exercise training reduces fat mass and adipose tissue inflammation. This can limit systemic inflammation (Fried et al. 1998; Harkins et al. 2004). In addition, muscular exercise increases muscle production of antiinflammatory myokines (Starkie et al. 2003). Regular exercise also increases vagal tone (Routledge et al. 2010), and stimulates the sympathetic nervous system, and this activates the antiinflammatory reflex (Utzschneider and Kahn 2006) and inhibits pro‐inflammatory cytokine production through catecholamine production (Ignatowski et al. 1996). Additionally exercise increases cortisol secretion with its potent antiinflammatory effects (Harbuz et al. 2003). Also, several studies (Flynn and McFarlin 2006) have demonstrated that exercise training can down regulate toll‐like receptor 4 that activates proinflammatory cascades (Takeda et al. 2003).

At the enzymatic level, treatment rats with DPPi for 6 weeks after ovariectomy significantly reduced the expression of SREBP1c transcription factor and ACC1 enzyme in the liver tissues and significantly increased PPAR*α* expression; all of which are essential regulators for hepatic fat metabolism, suppressing lipid accumulation in the liver and enhancing hepatic oxidation regaining the normal balance between the rate of synthesis and oxidation.

In a previous study carried out by Ding et al. (2006), it was reported that exendin‐4 significantly increases the expression of PPAR*α* mRNA in *ob/ob* mice. Also, Sitagliptin was found to decrease the expression of lipogenesis genes in the liver cells in wild type mice (Shirakawa et al. 2011).

Training exercise for 6 weeks after ovariectomy also reduced fat accumulation in the liver and it was shown to similarly regulate the enzymes involved in lipogenesis and lipid oxidation. Exercise training significantly added benefits to DPPi treatment as regard to lipid metabolism in the liver of ovariectomized rats, and achieved values insignificantly changed as compared with the control groups. Pighon et al. (2011) suggested that exercise training effects is comparable to estrogens in properly regulating liver enzymes and inflammatory biomarkers.

In addition to being the main transcriptional regulators of lipid and sterol biosynthesis (Shimomura et al. 1999), sterol regulatory element‐binding proteins (SREBPs) appear to have a role in regulation of autophagy (Sengupta et al. 2010).

In this study, it was found that autophagy was inhibited and its marker LC3 was significantly reduced 6 weeks after ovariectomy and was restored in the groups treated with DPPi and/or exercise training, suggesting another mechanism for DPPi and exercise in protecting liver cells from fat molecules accumulation.

This relation was supported in our work, since a significant negative correlation between LC3 and fat content in the liver tissue was recorded, suggesting a role of lipophagy process in regulation of fat droplet, prevention of their accumulation and development of fatty liver. The significant correlation also strongly suggests that restoring lipophagy is an important mechanism by which DPPi and exercise training can protect the hepatocytes in the ovariectomized rats.

Singh et al. (2009) reported that diminished formation of LC3‐II in the liver cells may be related to accumulation of fat droplets and development of fatty liver.

In accordance with our results, Lee et al. (2012) reported that exendin‐4 stimulated autophagy process in liver tissue obtained from high fat diet fed rats.

AMP‐activated protein kinase (AMPK) and silent mating type information regulation 2 homolog (sirtuin, SIRT) regulates energy homeostasis including fatty acid oxidation and lipogenesis (Canto and Auwerx 2009; Ruderman et al. 2010). An SIRT1 activator also has been shown to induce autophagy through AMPK activation (Puissant et al. 2010). GLP‐1 was reported to improve hepatic lipid metabolism by regulation of SIRT1/AMPK signaling. (Baggio and Drucker 2007) Also, Olivera et al. demonstrated that age‐related declines in the phosphorylation of AMPK and in the protein levels of SIRT1 in skeletal muscle can be reversed and largely improved by exercise. (Oliveira et al. 2014) Our findings indicate that the improvement in fat metabolism with DPPi and exercise was significantly correlated to changes in SIRT1/AMPK level suggesting that they are involved in the intracellular molecular activity of both DPPi and exercise in enhancing hepatocyte fat oxidation.

In the present study, it was evident that exercise training had a great impact on the liver function. A significant protection against damage was evident by the decreased liver enzyme values and histopathological examination. It also significantly reduced fat accumulation in the liver. The results recorded in the group of rats with exercise training were comparable to those reported after DPPi treatment in most aspects as compared to the untrained rats. Exercise training improved glycemic state, insulin sensitivity, modulated the inflammatory response with decreased expression of the proinflammatory markers and inhibited apoptosis, and regulated the enzymes of lipogenesis and lipid oxidation.

Adjuvant effect and maximum protection were offered by both exercise training and DPPi treatment with most achieved values returned to near control rats values.

## Conclusion

Findings from the present study confirm the derangement of fat metabolism in the ovariectomized rats and give a new insight on using incretin‐based therapy and changing life style by exercise training to improve liver fat metabolism through the modulation of the levels of enzymes responsible for lipid synthesis and oxidation acting through SIRT1/AMPK pathway, and enhancement of autophagy, improving insulin sensitivity and modulating the inflammatory response and apoptosis. DPPi treatment and physical activity synergistically achieved significant beneficial metabolic effects and offer full protection against NAFLD.

## Conflict of Interest

All authors of this paper certify that they have NO affiliations with or involvement in any organization or entity with any financial interest or nonfinancial interest in the subject matter or materials discussed in this manuscript**.**
